# Spatial genetic analysis reveals high connectivity of tiger (*Panthera tigris*) populations in the Satpura–Maikal landscape of Central India

**DOI:** 10.1002/ece3.432

**Published:** 2013-01-10

**Authors:** Sandeep Sharma, Trishna Dutta, Jesús E Maldonado, Thomas C Wood, Hemendra Singh Panwar, John Seidensticker

**Affiliations:** 1 Smithsonian Conservation Biology Institute, National Zoological ParkWashington, District of Columbia, 20013-7012; 2 Environmental Science & Policy Department, George Mason UniversityFairfax, Virginia, 22030-4444; 3 Department of Vertebrate Zoology, National Museum of Natural History, Smithsonian InstitutionWashington, District of Columbia, 20013; 4 Peace Institute Charitable TrustDelhi, India, 110091

**Keywords:** Central India, connectivity, non-invasive genetic analysis, *Panthera tigris*, spatial genetics, tiger

## Abstract

We investigated the spatial genetic structure of the tiger meta-population in the Satpura–Maikal landscape of central India using population- and individual-based genetic clustering methods on multilocus genotypic data from 273 individuals. The Satpura–Maikal landscape is classified as a global-priority Tiger Conservation Landscape (TCL) due to its potential for providing sufficient habitat that will allow the long-term persistence of tigers. We found that the tiger meta-population in the Satpura–Maikal landscape has high genetic variation and very low genetic subdivision. Individual-based Bayesian clustering algorithms reveal two highly admixed genetic populations. We attribute this to forest connectivity and high gene flow in this landscape. However, deforestation, road widening, and mining may sever this connectivity, impede gene exchange, and further exacerbate the genetic division of tigers in central India.

## Introduction

The tiger (*Panthera tigris*) is the largest extant cat species and has become an iconic conservation emblem for Asian forest ecosystems (Seidensticker 2010). Tigers historically ranged widely across Asia (Mazák 1981). By 2006, their occupancy had been reduced to 7% of their historical range, which was fragmented into 76 Tiger Conservation Landscapes (TCL) that were hypothesized to each contain one meta-population (Dinerstein et al. 2007). The Indian subcontinent has the largest number of TCLs (40, of which 11 are of global priority). These TCLs are home to 60% of wild tigers of the world (Sanderson et al. 2006) and the majority of this population is found in the alluvial flood plains of the Himalayan foothills, the Central Indian highlands, and the forests of Western Ghats (Jhala et al. 2011).

The “Central Indian highlands” is an important biogeographic province (Rodgers et al. 2002) and one of the six landscape complexes defined for tiger conservation in India (Jhala et al. 2011). It is occupied by 35% of India's tiger population, in 47% of India's remaining tiger habitat (Jhala et al. 2011). The Satpura–Maikal landscape is located in the central Indian highlands and categorized as a global-priority TCL for its potential to support long-term persistence of tigers (Dinerstein et al. 2007). It supports an estimated 12% of India's tiger population and contains 13% of India's tiger habitat (Jhala et al. 2011).

Tiger populations in India have been increasingly isolated over the last century due to habitat fragmentation and population decimation (Project Tiger-Tiger Task Force report 2005; Jhala et al. 2011). The genetic deterioration of insular populations can be prevented by gene exchange with neighboring populations by means of dispersing individuals and their successful breeding in the new population, thus maintaining a large and diverse gene pool (Bohonak 1999). Large terrestrial predators often exhibit limited genetic subdivision because they have high rates of dispersal-mediated gene flow (Wayne and Koepfli 1996).

In previous studies on the population genetics of tigers, various molecular methods revealed very low to moderate levels of genetic diversity in the tiger population of the Indian subcontinent. Shankaranarayanan et al. (1997) found that average heterozygosity was 0.28 in the RAPD (random amplified polymorphic DNA) analysis and 0.23 at three microsatellite loci in a study on the captive tigers in Indian zoos. Wentzel et al. (1999) also found very low levels of genetic variation in tigers at mitochondrial and nuclear genome segments. Luo et al. (2004) used 30 polymorphic microsatellite markers on Indian tigers and reported average observed heterozygosity of 0.524 (±0.039 SD), and average allele per locus of 3.5 (±1.22 SD). Mondol et al. (2009a) reported that Indian tigers are the most diverse among all tiger sub-species and have more than half of the extant genetic diversity in the species.

Recently, the tiger populations in India were classified into six landscape complexes on the premise that the habitat in each complex was contiguous in the recent past and the tigers living in them probably share a common gene pool (Jhala et al. 2011). However, this landscape taxonomy of tiger population sub-structuring was not based on an actual analysis of the genetic structure and gene flow of tiger meta-populations. Therefore, in this study, we aimed to investigate the patterns of genetic structure of a spatially extensive meta-population of tigers in central India (one of the six landscape complexes proposed by Jhala et al. 2011) to test the premise of this taxonomy.

In this paper, we present the results of our study of genetic diversity and fine-scale spatial genetic structure of tiger populations in the Satpura–Maikal landscape of central India using multilocus genotypic information from non-invasively collected samples. This landscape has lost more than 75% of its forest cover to farmlands and urbanization in the last 300 years (S. Sharma, T. Dutta, J. E. Maldonado, T. C. Wood, H. S. Panwar, J. Seidensticker, unpubl. data). This anthropogenic transformation of land may have posed a barrier to dispersal and gene flow among tiger populations and led to genetic subdivision. Therefore, we tested for genetic structure that might have been created by potential impediments to tiger dispersal and risks posed by the features in the existing corridors.

Our tests for genetic subdivision in this tiger meta-population were based on (1) assessing the genetic variation and estimating the population-level genetic difference by calculating and comparing *F*_ST_ and genetic distances between tiger populations, (2) by testing the pattern of isolation by distance (IBD) in this meta-population, and (3) using two different Bayesian clustering methods that utilize individual-based information to decipher underlying genetic subdivision patterns. In the last century, the tiger population in India has experienced a dramatic demographic decline that may have eroded the genetic variation and left a genetic signature of a bottleneck in this population. We used two different approaches to test for evidence of a genetic bottleneck in this meta-population.

## Materials and Methods

### Study area and sampling

**Figure 1:**
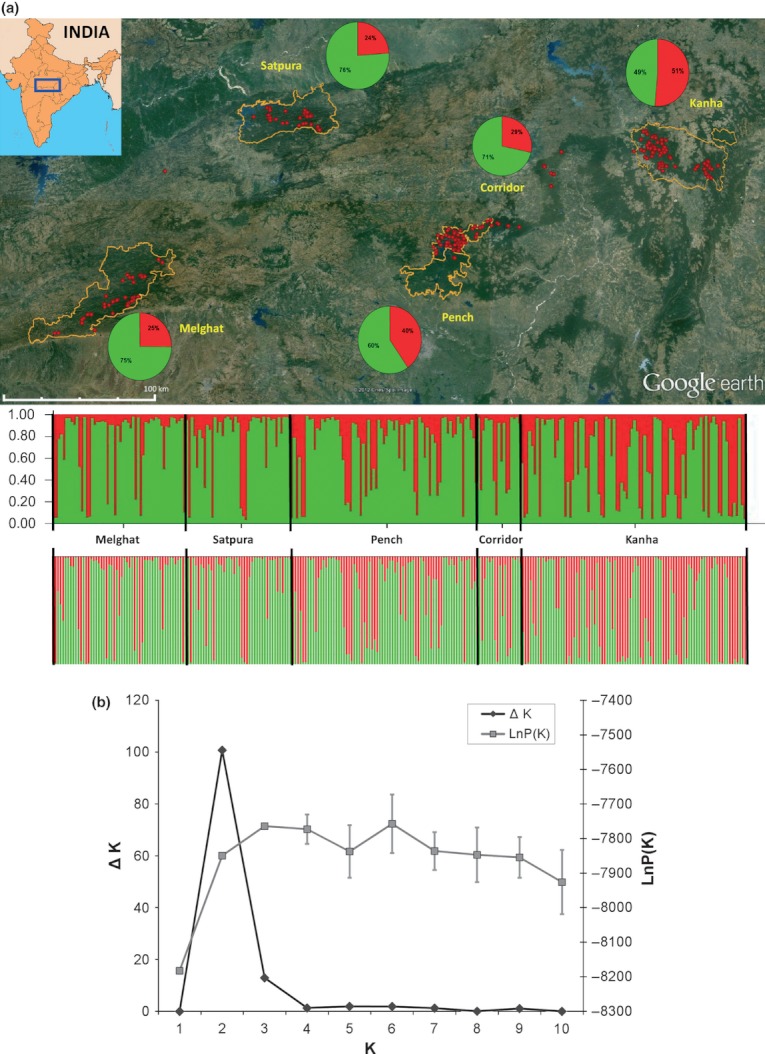
(a) Map of the Satpura–Maikal landscape with its location in India (inset). Red dots represent locations of individual tigers identified in each tiger reserve (Orange boundary) and corridors using multilocus genotype data. Pie charts show admixed proportions of genetic clusters for each tiger reserve, and correlate to the STRUCTURE bar plot (upper) and TESS bar plot (lower) at the bottom showing two admixed genetic populations for all sampling sites. Each line in the bar plot represents an individual tiger. (b) Magnitude of Δ*K* (rate of change in the log probability) and ln*P*(*K*) (posterior probability of the data) as a function of *K* (populations) detected two genetic clusters in the sampled populations.

Our study area in the Satpura–Maikal landscape in central India covers approximately 45,000 km^2^ (21.15–22.8°N and 76.5–81.05°E). The Satpura range is one of the oldest mountain ranges in the world and, together with the Vindhyachal range in the north and the Maikal range in the east, forms the catchments of the Narmada and the Tapti rivers and their tributaries (Krishnan 1982). Our study area consisted of the five major tiger reserves of this landscape: Kanha Tiger Reserve (Kanha), Bori-Satpura Tiger Reserve (Satpura), Pench MP (Madhya Pradesh) and Pench Mh (Maharashtra) Tiger Reserves (combined we refer to these two as Pench; as they are geographically connected to each other, but located in different states), and Melghat Tiger Reserve (Melghat), along with the forest corridors connecting these reserves. Kanha is connected to Pench and located toward the east of the landscape, while Melghat has a corridor with Satpura and lies to the west of the landscape (Fig. 1a). Tigers and their prey species were reported from these two corridors (Jhala et al. 2011). However, the intervening landscape matrix is composed of agricultural land and fragmented forest patches, interspersed with numerous small villages and towns (Jhala et al. 2011). Details describing the climatic and vegetation attributes of these tiger reserves are in Table S1.

During April–June 2009 and November 2009–May 2010, we conducted extensive surveys covering 15,000 km of forest trails and roads in these five tiger reserves and the corridors among them to collect fecal samples. We used systematic sampling inside the tiger reserves using a 10-km^2^ grid as the sampling unit (Fig. S1). All grids inside tiger reserves were sampled at least once except those that were completely occupied by dense human populations, barren-land, inaccessible terrain, or water bodies. We used stratified random sampling in corridors. Each corridor was stratified based on previous information about tiger occupancy (Jhala et al. 2008); grids with known occupancy were searched preferentially. We identified tiger fecal samples by their size and associated signs such as scrapes and pugmarks. We collected the outermost layer of the scat weighing approximately 5–10 g. Hair and claw samples were also collected opportunistically from trees marked by tigers and from kill sites. Only hairs that were found in a single clump were collected to avoid cross-individual contamination (see also error-checking methods below). Sample locations were recorded with a GPS unit (Garmin International, Inc., Olathe, Kansas) along with habitat information. Samples were preserved in 100% ethanol and stored at room temperature until further analysis.

### Laboratory methods

Genomic DNA from fecal samples was extracted using the QIAamp DNA Stool Mini Kit (Qiagen Inc. Valencia, California). We used DNeasy Blood and Tissue Kit (Qiagen Inc.) for DNA extraction from hair and claw samples. Negative controls were included in every batch of DNA extractions and downstream PCR procedures. Sample processing and genomic DNA extractions were conducted in an area exclusively dedicated to extraction and separate from DNA storage and PCR and post-PCR processing areas. Sterilized conditions were maintained during DNA extraction to prevent cross-contamination of samples.

In order to differentiate tiger fecal samples from those of sympatric leopards (*Panthera pardus*), whose feces can sometimes be confused with those of tigers, we screened each sample twice using tiger-specific mitochondrial DNA primer that amplifies a 164-bp fragment of the NADH5 region (Mukherjee et al. 2007), and leopard-specific mitochondrial DNA primer that amplifies a 130-bp fragment of the NADH4 region (Mondol et al. 2009c; Dutta et al. 2012). Those samples that were confirmed as tigers in duplicate runs along with a positive and negative control were then screened with the following panel of seven microsatellite loci: F42, F43, FCA279, FCA441, FCA628, FCA672 (Menotti-Raymond et al. 1999, 2005), and E7 (Bhagavatula and Singh 2006). These loci were specifically selected on the basis of their high amplification success, low error rates, and high PIC (polymorphic information content) to obtain multilocus genotypic data from fecal samples and have been used and optimized with tiger blood and tissue samples in previous studies (Xu et al. 2005; Bhagavatula and Singh 2006; Mondol et al. 2009b; Borthakur et al. 2011; Reddy et al. 2012a,b).

PCR reactions were performed in a 5-μL volume containing 2 μL QIAGEN Multiplex PCR buffer mix (Qiagen Inc.), 0.2 μmol/L labeled forward primer (Applied Biosystems, Carlsbad, California), 0.2 μmol/L unlabeled reverse primer, 10× Bovine Serum Albumin (New England Biolabs, Ipswich, Massachusetts), and 1- to 1.5-μL DNA template. The PCR conditions for microsatellites loci included an initial denaturation (94°C for 15 min); 45 cycles of denaturation (94°C for 30 sec), annealing (T_a_ for 45 sec), and extension (72°C for 45 sec); and a final extension (72°C for 30 min). We optimized the seven microsatellite loci to be conducted in two duplex (F42-F43 and FCA672-E7) and three separate (FCA279, FCA441, and FCA628) PCR reactions.

Each PCR reaction was repeated four times following the multi-tube approach (Taberlet et al. 1996) to reduce genotyping error and ensure reliability of genotypes. PCR products were separated using capillary electrophoresis on an ABI 3730XL sequencer and GeneScan™–500 LIZ® Size Standard (Applied Biosystems, Carlsbad, California), and scored on GeneMapper 4.1 (Applied Biosystems).

### Data analysis

#### Identification of unique genotypes and estimates of genotyping errors

A set of highly polymorphic loci with a low P_ID_ value (probability of pairs of individuals bearing an identical multilocus genotype) are necessary for obtaining more precise estimates of population structure (Waits et al. 2001). We used CERVUS 3.0.3 (Kalinowski et al. 2007) to compute P_ID_ and P_ID (SIBS)_ and to match identical genotypes. Only those samples that amplified at least three times of four PCRs during multi-tube replication were used to create consensus genotype. A criterion of five of seven matching loci was used to compare each multilocus genotype and identify individuals, because the P_ID_ value was low enough (unbiased P_ID_ = 1.237e−07 and P_ID (SIB)_ = 5.584e−03) even when using the five most polymorphic loci.

Genotyping errors are an inherent problem associated with studies that use non-invasively collected samples such as feces and hair (Taberlet et al. 1996; McKelvey and Schwartz 2004). They can produce erroneous outcomes and hence should be carefully computed, eliminated, and reported in any study based on non-invasive DNA sampling. We used various approaches implemented in different programs to estimate and remove genotyping errors. We used the “examining bimodality” (EB) test and the “difference in capture history” (DCH) test (McKelvey and Schwartz 2004; Schwartz et al. 2006) implemented in the program DROPOUT (McKelvey and Schwartz 2005) to detect and assess error rates (dropout, false allele, and scoring error). We also used MICRO-CHECKER 2.2.3 (Van Oosterhout et al. 2004) to search for loci with short allele dominance and stuttering error. We used FreeNA (Chapuis and Estoup 2007) to detect and estimate null allele frequencies for each locus and population. This software uses the Expectation Maximization (EM) algorithm (Dempster et al. 1977) and has been shown to perform better than other conventional estimators (Chapuis and Estoup 2007).

In addition, in order to ensure that hair samples from each clump were collected from a single individual, we checked for the presence of multiple alleles (more than two alleles) for every locus typed to address the potential problem of creating false genotypes that may arise as a result of genotyping hairs from multiple individuals. We did not detect any consistent pattern of multiple alleles for any of the loci from any of the hair clumps.

#### Estimation of genetic variation and population subdivision

Genetic diversity for all study sites was measured as alleles per locus (A), allelic richness (AR), observed heterozygosity (*H*_o_), and expected heterozygosity (*H*_e_) using FSTAT 2.9.3.2 (Goudet 1995). We checked all loci at the global and population level for deviations from Hardy–Weinberg equilibrium (HWE) using an exact test (Guo and Thompson 1992) with 10,000 dememorization steps, 1000 batches, and 10,000 iterations per batch in GENEPOP 3.4 (Raymond and Rousset 1995). Linkage disequilibrium (LD) among all loci pairs was also assessed in GENEPOP. In multiple comparisons of significance test for departures from HWE and LD, Bonferroni corrections were applied to a significance value (α = 0.05; Rice 1989).

*F* statistics (*F*_ST_: population-level genetic difference among study sites; *F*_IS_: inbreeding coefficient) were estimated using ARLEQUIN 3.11 (Excoffier et al. 2005). We calculated an improved measure of genetic distance *D*_EST_ (Jost 2008), using SMOGD 1.2.5 (Crawford 2010), and also measured the Cavalli-Sforza and Edwards' genetic distance (Cavalli-Sforza and Edwards 1967) for all study site pairs using FreeNA. We also created a neighbor-joining tree using the Cavalli-Sforza and Edwards' genetic distance and estimated the *R*^2^ value that estimates the degree of fit of a tree to a matrix of genetic distance in the program TreeFit 1.2. (Kalinowski 2009). We also ran 10,000 bootstrap iterations to measure the statistical confidence in tree topology in TreeFit 1.2.

Frantz et al. (2009) suggest determining if a pattern of IBD exists before using any clustering methods to test for population sub-structuring. Therefore, we tested for IBD using the sub-program ISOLDE (Rousset 2000) in GENEPOP. This sub-program implements a Mantel's test to check the correlation between genetic distance (*F*_ST_) and the geographical distance matrices. Because the Euclidian distance between study sites is not a true representation of their connectivity, we used the distance measured through forest connectivity between all pairs of study sites on Google Earth (Ver.6.0.3.2197) as the geographical distance.

#### Detection of genetic structure

We used two different Bayesian clustering methods to detect any pattern of genetic structure in this landscape. The first method as implemented in the program STRUCTURE 2.3.2 (Pritchard et al. 2000) uses Markov chain Monte Carlo (MCMC) method to estimate *P*(*X*|*K*), the posterior probability of the multilocus genotypic data from individual samples to fit into a number of predefined clusters (*K*), so as to minimize the deviations from HWE. We conducted 10 independent runs for *K* ranging from 1 to 10 using admixture model and correlated allele frequencies, using a burn-in for 100,000 steps, followed by 100,000 steps for MCMC runs for data collection. The optimal value of *K* was selected using the posterior probability of the data for a given *K* (ln*P*(*K*)) and the second-order rate of change of log probability of the data between successive values of *K* (Δ*K*; Evanno et al. 2005) in the program STRUCTURE HARVESTER v0.6.8 (Earl and vonHoldt 2011). We also calculated and plotted the cumulative admixture proportion of inferred *K* in each tiger population using inferred fractional membership of each individual in genetic clusters (*Q*).

STRUCTURE is known to perform poorly and incorrectly assign individuals when *F*_ST_ values are low (*F*_ST_ < 0.05) among populations (Latch et al. 2006; Chen et al. 2007). Spatial models are generally known to perform better than non-spatial models, and are more efficient than non-spatial models at low *F*_ST_ values. Spatial models also outperform non-spatial models such as STRUCTURE in detecting clines at low *F*_ST_ values (Chen et al. 2007; François and Durand 2010). The second method we used was a spatially explicit clustering method implemented in the program TESS 2.3 (Chen et al. 2007) that builds a spatial individual neighborhood network using the Voronoi tessellation. The prior distribution of cluster labels is calculated using hierarchical mixture models. TESS uses the spatial information along with multilocus genotypic data from individuals to define population structure without using predefined population information. TESS performs better than STRUCTURE in detecting the number of hidden genetic populations and also works well at moderate levels of admixture (Chen et al. 2007; François and Durand 2010) and therefore it should be used along with STRUCTURE to address inferences of spatial population structure (Chen et al. 2007). We used the admixture model with both programs. The admixture model works on the premise that the individuals sampled are an admixture of *K* putative parental populations that may be unavailable for study. Furthermore, a comparison of various spatially explicit, Bayesian clustering models found that admixture models are robust in identifying diverging sub-populations (Falush et al. 2003; François and Durand 2010).

We ran the TESS analysis for 10,000 burn-ins followed by 50,000 run-in sweeps for *K* (2–10). We used both admixture models (BYM and CAR) for this analysis (Chen et al. 2007). We used the Deviance Information Criterion (DIC) to estimate the number of clusters (genetic populations) and to compare the performance of various models used in the analysis (Spiegelhalter et al. 2002).

#### Detection of genetic bottleneck

We were interested to assess if this meta-population shows a genetic signature of undergoing a severe demographic contraction. To test this, we used the program BOTTLENECK (Piry et al. 1999) and also calculated M-Ratio (Garza and Williamson 2001) in ARLEQUIN 3.11. The Program BOTTLENECK uses three different quantitative tests (sign-test, standardized differences test, and Wilcoxon signed-rank test) and one qualitative test (mode-shift test) to compare the distribution of the heterozygosity expected from the observed number of alleles (*k*), given the sample size (*n*) for each population sample and for each locus under the assumption of mutation-drift equilibrium (Cornuet and Luikart 1996). We used a two-phased mutation model (TPM) with 90% SMM (stepwise mutation model) and implemented Wilcoxon signed-rank test (which is the most powerful and works with a few loci) along with a mode-shift test to detect bottlenecks in various tiger populations and the putative genetic clusters. The M-ratio test compares the number of alleles (*k*) with the allelic size range (*r*). In a bottlenecked population as rare alleles are lost, *k* is reduced faster than *r*, and therefore a low M-ratio relative to a critical value (0.68) indicates population bottleneck (Garza and Williamson 2001). The M-ratio test is considered to be a more sensitive measure of population bottleneck than the heterozygosity excess tests performed in BOTTLENECK program (Piry et al. 1999).

## Results

### Sampling summary

We collected 1411 felid fecal samples, 66 hair samples, and 4 claw samples from the entire study area during two sampling sessions in the years 2009–2010. We identified 463 tiger-positive samples and 287 leopard-positive samples (Dutta et al. 2012, in press), of which 372 amplified for more than five microsatellite loci and more than 75% success in multi-tube replication. The identity analysis as performed using CERVUS identified 273 individual tigers in the study area with 99 recaptures; this includes one individual from the Satpura–Melghat corridor and 17 individuals from the Kanha–Pench corridor (Table 1). For subsequent analysis, we considered tigers from the Kanha–Pench corridor as a separate population named “Corridor.” The minimum number of individual tigers we identified in each tiger reserve using genetic analysis is similar to those found by the population estimation exercise conducted by the National Tiger Conservation Authority (NTCA) and Wildlife Institute of India (WII) using a suite of methods including mark-recapture-based estimation, prey biomass, and occupancy-based modeling (Jhala et al. 2011; Table 1).

**Table 1:** Information about sampling and genotyping success, individual identified, and comparison with the population estimates by National Tiger Conservation Authority-Wildlife Institute of India (NTCA-WII) in 2010 for all sampling sites

Sampling location	Non-invasive samples collected	Tiger-positive sample	Selected genotypes^1^	Individual identified	NTCA-WII estimates
Corridor-1	43	20	17	17	NA
Corridor-2	3	1	1	1	NA
Kanha TR	604	182	131	89	60 (45–75)
Pench TR	357	123	104	73	65 (53–78)
Satpuda TR	246	62	54	41	43 (42–45)
Melghat TR	228	75	65	52	35 (30–39)
Total	1481	463	372	273	203 (175–237)

1 A criterion of amplification of at least five loci of seven was used to select these genotypes.

### Identifying unique genotypes and estimation of genotyping error

The cumulative discriminatory power of seven loci to identify individuals was very high (unbiased P_ID_ = 1.023e−09 and P_ID (SIB)_ = 7.411e−04). Genotyping error (dropout and false allele) and scoring error were detected, calculated, and removed using the EB test and the DCH test in the program DROPOUT (Table 2; Fig. S2). The mean genotyping error rates were low (false allele: 0.006 ± 0.003 SD; dropout: 0.011 ± 0.008 SD; scoring error: 0.008 ± 0.004 SD). No evidence of stuttering error and short allele dominance was found using MICROCHECKER. Fewer than 8% null alleles were detected on average, and this was not consistent for any loci.

**Table 2:** Locus-specific information on repeats, size range, chromosomal assignments, and genotyping error rates

Locus	Repeat motifs	Size range (bp)	Chromosomal assignment	False allele	Dropout	Scoring error
F42	Tetra-nucleotide	207–255	A1	0.006	0.026	0.007
F43	Di-nuclotide	106–132	C2	0.001	0.005	0.009
FCA279	Di-nuclotide	85–113	C1	0.007	0.008	0.009
FCA441	Tetra-nucleotide	101–125	D3	0.006	0.012	0.001
FCA628	Di-nuclotide	91–121	D2/E3	0.009	0.012	0.005
FCA672	Di-nuclotide	82–110	F2	0.01	0.001	0.013
E7	Di-nuclotide	133–159	NA	0.005	0.015	0.012
Mean				0.006	0.011	0.008
SD				0.003	0.008	0.004

### Genetic diversity and population subdivision

**Figure 2:**
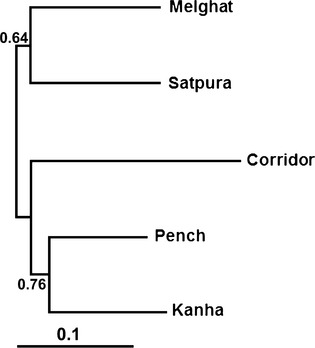
The neighbor-joining tree showing Kanha-Pench TRs and Corridor in one group and the Satpura–Melghat TRs in another group. The numbers close to the nodes are bootstrap support.

All seven loci were polymorphic across all study sites (Table 3). The genetic diversity measures A and AR were 12.4 (±3, SD) and 7.76 (±1.96, SD), respectively. The mean *H*_o_ and *H*_e_ for all the study sites were 0.65 (±0.09, SD) and 0.81 (±0.05, SD). Four loci (F42, FCA279, FCA441, and FCA628) were found to be out of HWE in the global test, after Bonferroni corrections, but not consistently when samples were analyzed by study sites as separate populations. Two loci in Melghat and Pench, one in Satpura, three in Corridor, and no loci in Kanha were out of HWE (Table 3). Details of population-specific genetic diversity are given in Table 3. No significant linkage disequilibrium was found in pairwise loci comparisons. The mean *F*_ST_ value among all study site pairs was low (0.013, ±0.006 SD). Similarly, mean *D*_EST_ values and mean genetic distance were (0.035 ± 0.018 SD) and (0.25 ± 0.003 SD), respectively (Table 4 and 5), which shows a very weak population subdivision. The neighbor-joining tree clustered the Kanha–Pench TR and Corridor in one group and the Satpura–Melghat TR in another group with a high support value (*R*^2^ = 0.96) for the tree (Fig. 2). This suggests that the tiger meta-population in the Satpura–Maikal landscape is subdivided into two groups that have close genetic affinities.

**Table 3:** Measures of genetic diversity at seven microsatellite loci in five tiger populations (*n* = 273) of the Satpura–Maikal landscape

			Kanha (n = 89)					Cooridor (n = 17)					Penchb (n = 73)		
												
Locus	A	AR	Ho	He	F_IS_	A	AR	Ho	He	F_IS_	A	AR	Ho	He	F_IS_
F42	9	6.8	0.5	0.76	0.23	6	6	0.64	0.82	0.35	12	6.9	0.39	0.79^2^	0.51
F43	9	6.09	0.76	0.77	0.09	7	6.37	0.69	0.76	0	8	6.32	0.74	0.78	0.05
FCA279	11	7.89	0.69	0.88	0.12	10	9.27	0.76	0.86	0.23	13	8.43	0.63	0.86	0.27
FCA441	7	5.08	0.65	0.85	0.36	7	6.82	0.49	0.76^2^	0.24	6	5.08	0.65	0.77	0.16
FCA628	11	8.13	0.73	0.8	0.23	8	7.36	0.65	0.85^2^	0.08	12	7.62	0.62	0.79	0.21
FCA672	12	8.59	0.71	0.85	0.22	9	8.01	0.68	0.87^2^	0.18	13	8.42	0.88	0.83	-0.05
E7	8	5.19	0.65	0.79	-0.16	6	5.88	0.8	0.69	0.19	13	7.3	0.62	0.77^2^	0.2
Mean	9.57	6.82	0.67	0.81	0.16	7.57	7.10	0.67	0.80	0.18	11.00	7.15	0.65	0.80	0.19
SD	1.81	1.43	0.09	0.05	0.16	1.51	1.22	0.10	0.07	0.11	2.83	1.19	0.15	0.03	0.18

			Satpuda (n = 42)^1^					Melghat (n = 52)					All population (n = 273)		
											
Locus	A	AR	Ho	He	F_IS_	A	AR	Ho	He	F_IS_	A	AR	Ho	He	F_IS_
F42	10	8.31	0.39	0.86^2^	0.54	9	7.53	0.5	0.87^2^	0.43	12	8.08	0.49	0.82^2^	0.4
F43	8	6.05	0.63	0.75	0.16	7	6.14	0.63	0.67	0.06	10	5.45	0.69	0.75	0.07
FCA279	11	8.57	0.63	0.86	0.27	14	8.71	0.73	0.88	0.18	15	9.4	0.69	0.87^2^	0.19
FCA441	5	4.83	0.56	0.75	0.26	7	5.46	0.58	0.79^2^	0.27	7	5.93	0.59	0.79^2^	0.26
FCA628	9	7.52	0.66	0.85	0.23	14	8.54	0.82	0.89	0.08	14	9.96	0.7	0.84^2^	0.18
FCA672	13	8.2	0.73	0.81	0.09	13	8.81	0.75	0.88	0.15	15	9.57	0.75	0.85	0.1
E7	12	8.49	0.65	0.84	0.23	10	6.96	0.59	0.69	0.14	14	5.93	0.66	0.76	0.08
Mean	9.71	7.42	0.61	0.82	0.25	10.57	7.45	0.66	0.81	0.19	12.43	7.76	0.65	0.81	0.18
SD	2.69	1.44	0.11	0.05	0.14	3.10	1.33	0.11	0.10	0.13	2.99	1.96	0.09	0.05	0.12

A, alleles per locus; AR, allelic richness; *H*_o_, observed heterozygosity; *H*_e_, expected heterozygosity; *F*_IS_, inbreeding coefficient.

1 One individual tiger identified from Satpura–Melghat corridor is placed in Satpura population based on its inferred fractional membership in STRUCTURE analysis.

2 Significant values for Hardy–Weinberg equilibrium following Bonferroni correction (α = 0.5).

**Table 4:** Pairwise *F*_ST_ (below diagonal) and *D*_EST_ (above diagonal) values for the five study sites

	Corridor	Kanha	Pench	Satpura	Melghat
Corridor	–	0.035	0.005	0.040	0.048
Kanha	0.002^1^	–	0.025	0.054	0.045
Pench	0.004	0.008^1^	–	0.036	0.061
Satpura	0.009	0.014^1^	0.015^1^	–	0.008
Melghat	0.019^1^	0.015^1^	0.025^1^	0.008^1^	–

1 *F*_ST_ values are significant (α = 0.05).

**Table 5:** Pairwise Cavalli-Sforza and Edwards' genetic distance (below diagonal) and geographical distance (distance through forest connectivity in km) (above diagonal) values for the five study sites

	Corridor	Kanha	Pench	Satpura	Melghat
Corridor	–	90	45	230	270
Kanha	0.311	–	145	275	370
Pench	0.269	0.205	–	115	155
Satpura	0.309	0.246	0.218	–	125
Melghat	0.294	0.236	0.251	0.243	–

There was no significant correlation (*r* = 0.06, *P* = 0.26) between geographical distance and the genetic distance matrices using a Mantel's test in ISOLDE sub-programs implemented in GENEPOP. This shows an absence of IBD in this meta-population.

### Genetic structure

**Figure 3:**
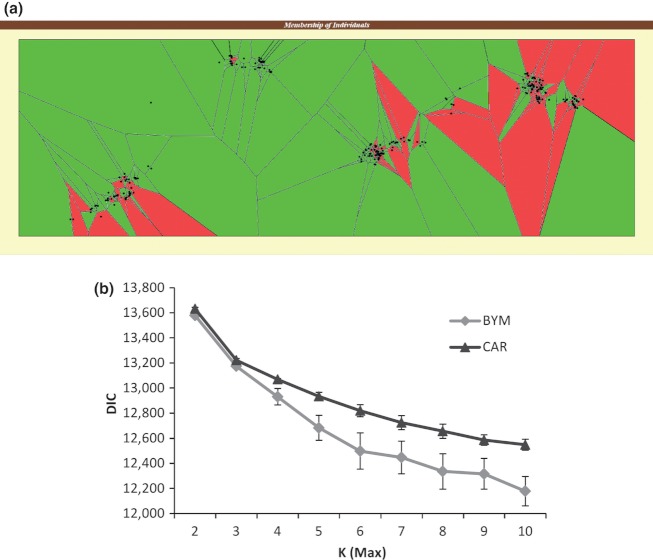
(a) Output from spatial clustering analysis in TESS, showing two genetic populations with predicted membership and location of each sampled individual (Black dots). Each cell (Dirichlet or Voronoi cell) and its color correspond to the predicted membership of that individual in a genetic population. The black lines are boundary or common edge of each cell and are exactly in the middle of the distance from all the nearest points. (b) Selection of optimal number of genetic clusters on the basis of DIC criterion for both models (BYM and CAR), detecting two genetic populations. Error bars are standard deviation.

The genetic clustering analysis using STRUCTURE indicated weak genetic structure and revealed two admixed genetic populations as inferred by the ln*P*(*K*) and Δ*K* method (Fig. 1b). The admixture proportions (*Q*) were variable for different study sites (Fig. 1a). The admixed proportions from the two genetic population (A, B) in all the study sites were as follows: KTR (49%, 51%), PTR (60%, 40%), STR (76%, 24%), MTR (75%, 25%), and Corridor (71%, 29%). These results were corroborated by spatially explicit results from TESS (Fig. 1a), which estimated two genetic clusters for both models using the DIC criterion, with admixed populations in Kanha, Corridor, Pench, and Melghat, while Satpura is relatively differentiated (Fig. 3a and b).

We did not detect any indication of heterozygote excess under mutation–drift equilibrium in any of the five study sites. The allele frequency distribution in the mode-shift test had an L-shaped distribution indicating no bottleneck event, which was further confirmed by non-significant values for both two-tailed and one-tailed Wilcoxon signed-rank test. The average M-ratio for all study sites was 0.73 (±0.08, SD), which was higher than the threshold value of 0.68 found in a meta-analysis (Garza and Williamson 2001).

## Discussion

This is the first study to attempt to assess the fine-scale spatial genetic structure of a tiger population at a landscape scale. We used seven highly polymorphic microsatellite loci to obtain genotypic information from non-invasive samples to measure the genetic structure of the tiger meta-population in the Satpura–Maikal landscape in central India.

The overall genetic variation of the entire tiger meta-population in our study area was high with allele numbers per locus ranging from 7 to 15 and high heterozygosity (*H*_e_ = 0.81 ± 0.05 SD). The within-population genetic variation in all of our five study sites was similarly high (*H*_e_∼0.80), showing no loss of genetic diversity at the landscape scale. Although measures of genetic diversity of tiger populations are available from Western Ghats (*H*_e_ = 0.76 ± 0.07 SD, mean number of alleles per locus 8.6 ± 2.27 SD; Mondol et al. 2009b), from south Eastern Ghats (*H*_e_ = 0.58; Reddy et al. 2012b), from Western India (*H*_e_ = 0.76; Reddy et al. 2012a), and from the Brahmaputra flood plain (*H*_e_ = 0.63 ± 0.09 SD, mean number of alleles per locus 5.13 ± 1.73 SD; Borthakur et al. 2011), a direct comparison with these results cannot be made because these studies used a different set of microsatellite loci. A country-wide study of population genetic structure of tigers using mitochondrial markers found the lowest nucleotide diversity in the central Indian tiger population (Sharma et al. 2009), but a subsequent study found high variation (0.70 ± 0.16 SD, and number of alleles: 12.4 ± 3.6 SD) using five microsatellite loci in a wide-scale analysis of Indian tigers (Mondol et al. 2009a). Our results based on seven microsatellite loci suggest that tigers in central India are genetically diverse.

We used population- and individual-based approaches to assess the levels of genetic structure in the tiger population in this landscape. Population-based tests revealed low but significant population subdivision in this landscape. The tiger reserve pairs that are inter-connected by contiguous forest corridors (Kanha–Pench and Satpura–Melghat, see Fig 1a) have very low *F*_ST_ values (*F*_ST_ = 0.008, ±0 SD), and the tiger reserve pairs located most distant from each other and lacking a forest corridor (Kanha–Satpura, Kanha–Melghat, Pench–Satpura, and Pench–Melghat) have somewhat higher *F*_*ST*_ values (*F*_ST_ = 0.017, ±0.005 SD). The *D*_EST_ values showed similar results (Table 4). Tigers are known to disperse long distances and we suggest that tiger dispersal through the forested corridor connecting these tiger reserves has maintained gene flow and prevented genetic subdivision in this landscape. Furthermore, our results suggest that these populations have not undergone a population bottleneck in the recent past.

We detected signatures of two genetic populations in our study area using STRUCTURE and TESS with the admixture model. There is no sharp boundary between these two populations and they are highly admixed as seen in the STRUCTURE plot (Fig. 1a).

The weak genetic structure that we detected in the population- and individual-based analyses in tiger meta-population of the Satpura–Maikal landscape can be explained by the history of land-use change in this landscape. The entire landscape was largely undisturbed and forested until the very recent past (∼150 years ago) and was known to be occupied by tigers (Forsyth 1871; Rangarajan 1996). By the late 19th century, the flat alluvium along with the Narmada basin had been gradually deforested and converted to agriculture, but the highland forests of the Satpura and Vindhyachal ranges were still inhospitable to people (or to agriculture), maintaining the connectivity among tiger-occupied forests. The demand for wood to construct railroads in India during the early British era (1859–1878) escalated the denudation of these central highland forests, leading to fragmentation of tiger habitat (Rangarajan 1996). Historically, many forest patches in the central highland forests were privately owned. After the independence of India in 1947, rights in private forests were abrogated as a measure of land reform. Subsequently, most of these state-resumed forests were encroached, deforested, and riddled with farm-pockets where there was better soil (Rangarajan 1996). This compromised the corridor value, especially where private forest areas were large. The tiger population plummeted in post-independence India due to organized sport hunting, until it was banned in 1970, and inviolate areas for tigers were notified under the Wildlife Protection Act in 1972. The tiger population grew from 1972 until the 1980s (Panwar 1987). Thereafter, the tiger population has been continually depleted by burgeoning poaching pressure (Mills and Jackson 1994; Kumar and Wright 1999; Banks et al. 2006) and habitat loss (Jhala et al. 2011). The present proliferation of roads, rail lines, mining, urbanization, and other forms of development to sustain India's economic growth through the remaining forest tracts connecting protected areas jeopardizes the persistence of the tiger populations (Project Tiger 2005; Jhala et al. 2011).

The tiger meta-population in the Satpura–Maikal landscape has high levels of genetic variation, very low *F*_ST_ values, and a weak genetic structure. If the anthropogenic forest fragmentation is <100–200 years old in this landscape, its effect on pairwise *F*_ST_ values of this tiger meta-population will not be detected for yet some time. The reason for this is that the *F*_ST_ statistic has a lag time of about 200 generations after the effect on gene flow can be detected due to the formation of a new barrier (Landguth et al. 2010). However, IBD tests have a much shorter lag time (1–15 generations) to detect a new barrier (Landguth et al. 2010), but for a highly vagile species like the tiger, we did not expect an IBD at the spatial scale of our study landscape. The individual-based clustering algorithm reflects the most recent changes in the landscape and their effects on allelic variation of the population. We detected the presence of two highly admixed genetic populations in this landscape with a rigorous analysis using both spatial and non-spatial models. This indicates either a genetic division of a panmictic population in the recent past or a zone of contact between two distinct genetic populations. Further sampling from tiger populations located adjacent to the Satpura–Maikal tiger meta-population may provide more information on this. We suggest replication of population genetics study like ours in other TCLs. The contemporary classification of TCLs is based on tiger habitat occupancy and observed forest connectivity as a surrogate to assess the extent of genetic connectivity of tiger populations (Wikramanayake et al. 2011). A similar landscape-level genetic study in these TCLs will provide more pragmatic information about the connectivity, functionality, and extent of the tiger meta-populations in these landscape units. It will also help in designing biologically meaningful tiger conservation and management units.

Our study establishes the fact that genetic populations exceed the confinement of source populations and tigers require large landscapes consisting of breeding populations interconnected with forest corridors, for their long-term persistence. Tiger conservation efforts should be targeted at a landscape-scale level (Wikramanayake et al. 2011) rather than merely focused on the source populations (Walston et al. 2010).

We found that the tiger meta-population in the Satpura–Maikal landscape has low levels of genetic structure. Genetic subdivision was low between tiger reserves that were connected with forest corridors, thus lending support toward the functionality of this connectivity. The future of this tiger meta-population relies on implementing an effective policy that includes further protecting the tiger populations and their habitat in the tiger reserves and forested corridors that connect them.

## Supporting Information

**Figure S1.** Satpura–Maikal landscape in central India. Orange polygons are tiger reserves; blue lines are sampling tracks; red dots are tiger-positive samples (*n* = 463). The cell size of the overlaid grid was 10 km^2^. This map also shows locations of major cities and major highways (light yellow lines).

**Figure S2.** The bar plots for “examining bimodality” (EB) test (in blue) and “difference in capture history” (DCH) test (in green) with our data. The top two plots are before removal of error and show the loci with their error rates in the DCH plot. The bottom plots are after removal of the error as evident from the DCH plot that shows no new individual added at any loci after the error removal.

**Table S1.** Information about climatic and vegetation attributes of tiger reserves in the Satpura–Maikal landscape.
