# Supplementary material for: Spatial genetic analysis reveals high connectivity of tiger (Panthera tigris) populations in the Satpura–Maikal landscape of Central India
Source: Ecol Evol. 2013 Jan 10;3(1):48–60. doi: 10.1002/ece3.432 (PMC3568842; doi:10.1002/ece3.432)
Supplement: Supplementary file 1 [file ece30003-0048-SD1.pdf]

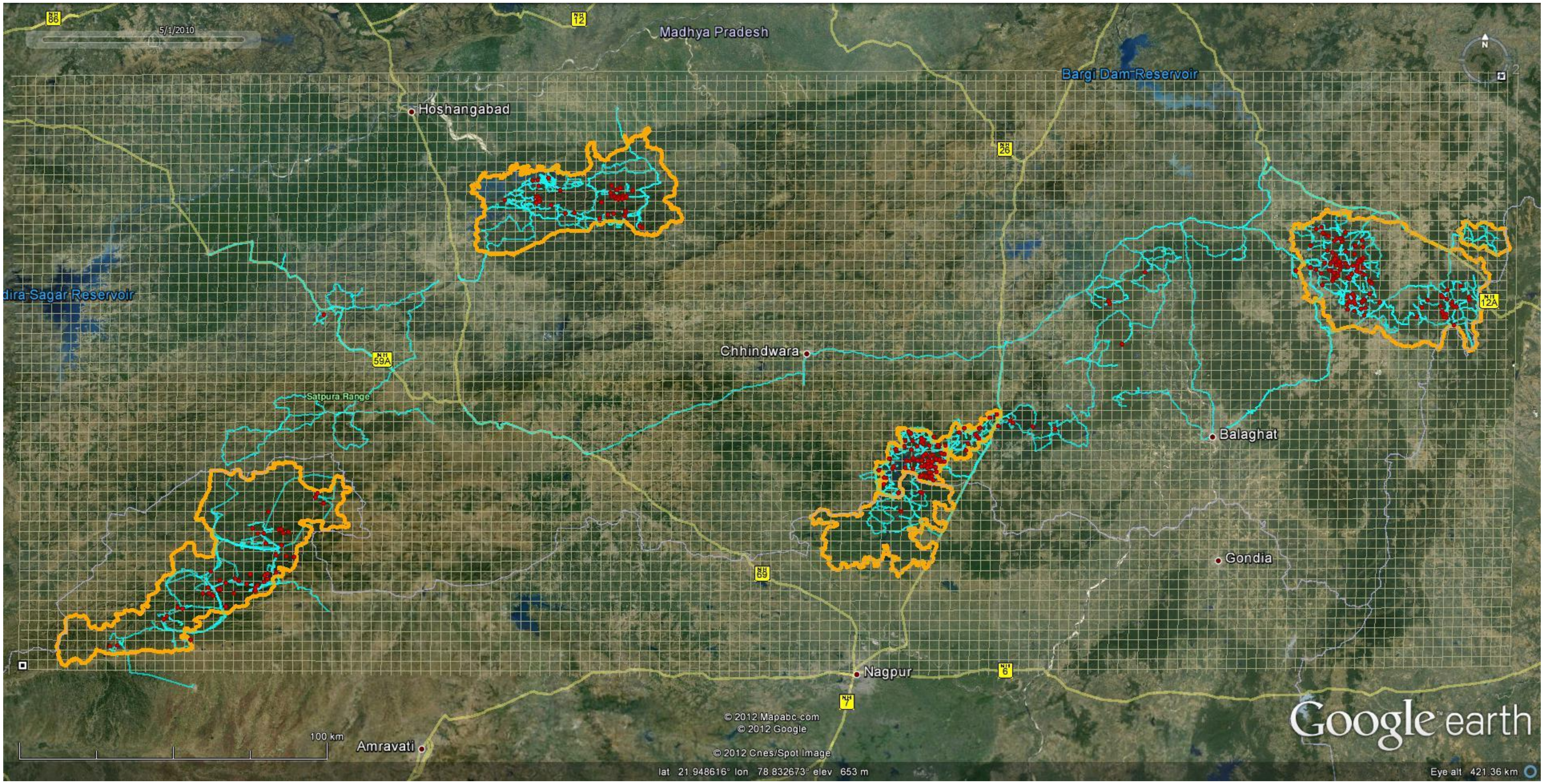

Figure S 1: Satpura-Maikal landscape in central India. Orange polygons are tiger reserves, blue lines are sampling tracks, red dots are tiger positive samples (n = 463). The cell size of the overlaid grid was 10 km^2. This map also shows locations of major cities and major highways (light yellow lines).
