# Supplementary figures and images for: Spatial genetic analysis reveals high connectivity of tiger (Panthera tigris) populations in the Satpura–Maikal landscape of Central India
Source: Ecol Evol. 2013 Jan 10;3(1):48–60. doi: 10.1002/ece3.432 (PMC3568842; doi:10.1002/ece3.432)

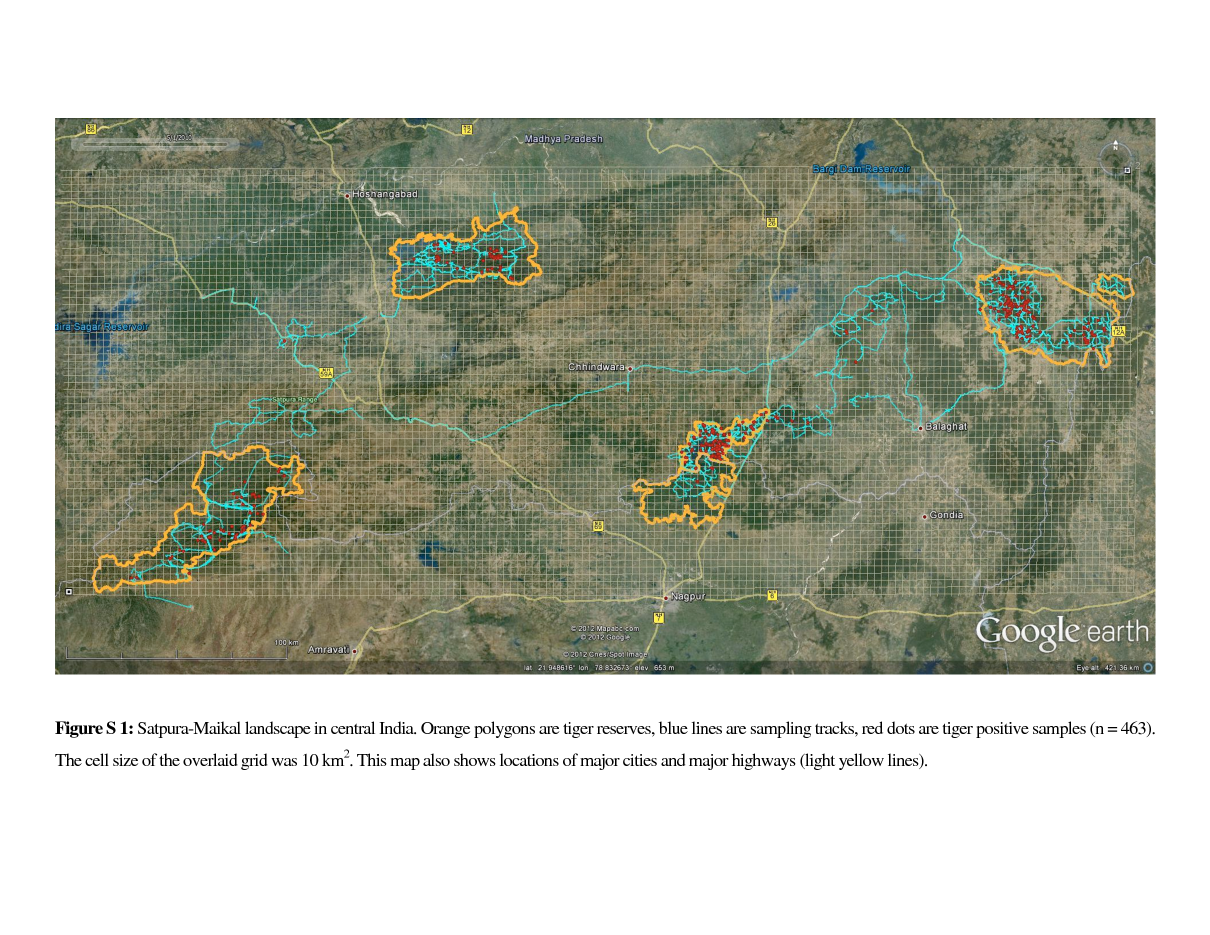

Supplement: Supplementary file 2 [file ece30003-0048-SD4.png]

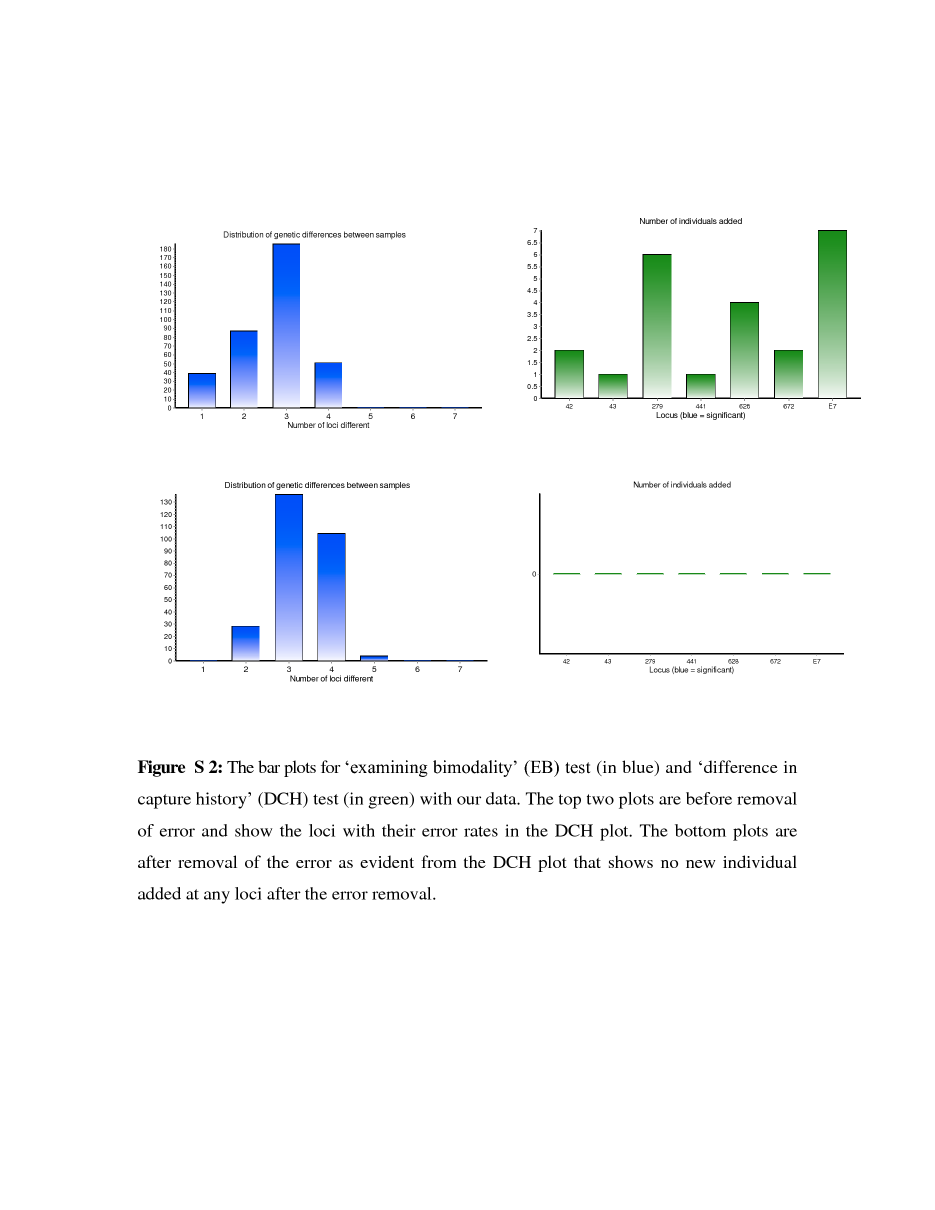

Supplement: Supplementary file 5 [file ece30003-0048-SD5.png]

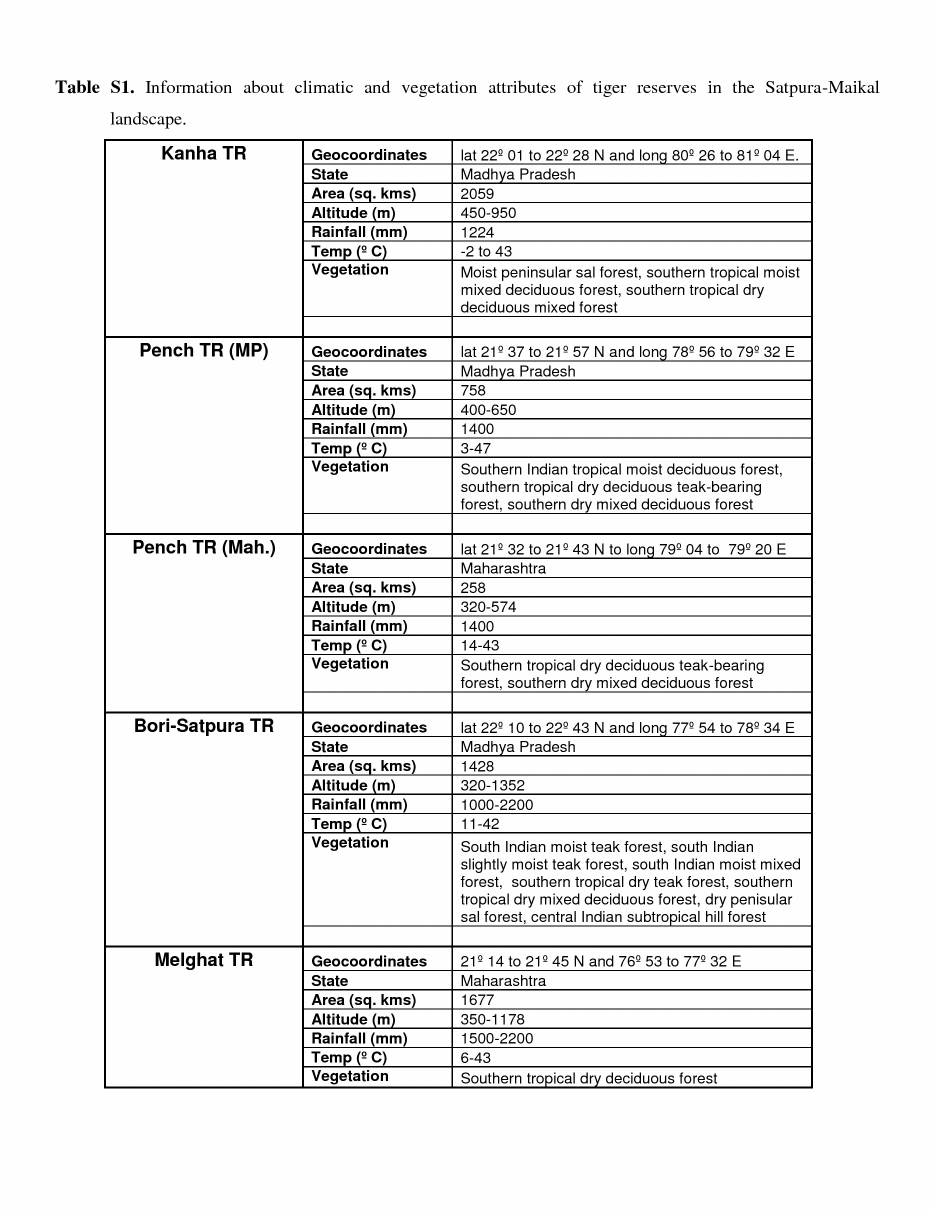

Supplement: Supplementary file 8 [file ece30003-0048-SD6.png]
