# Supplementary material for: Spatial genetic analysis reveals high connectivity of tiger (Panthera tigris) populations in the Satpura–Maikal landscape of Central India
Source: Ecol Evol. 2013 Jan 10;3(1):48–60. doi: 10.1002/ece3.432 (PMC3568842; doi:10.1002/ece3.432)
Supplement: Supplementary file 4 [file ece30003-0048-SD2.pdf]

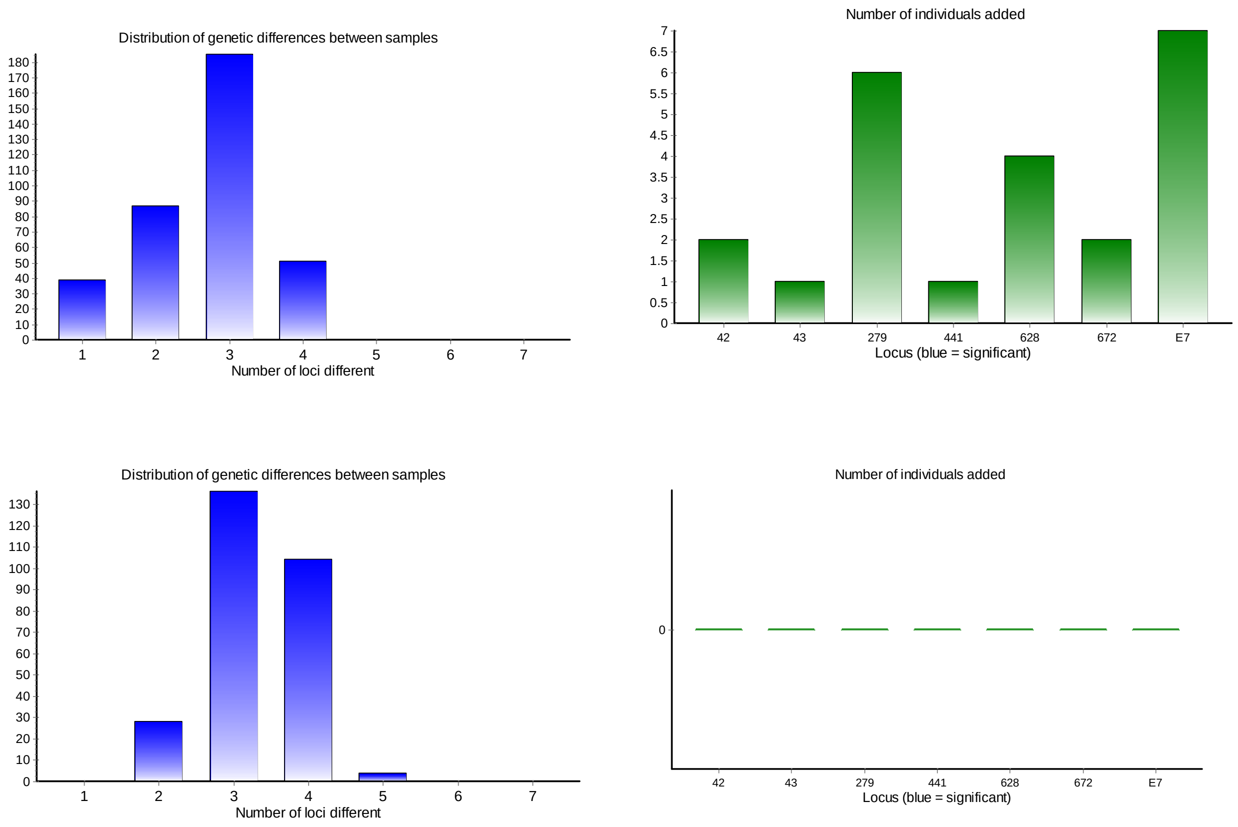

Figure S 2: The bar plots for ‘examining bimodality’ (EB) test (in blue) and ‘difference in capture history’ (DCH) test (in green) with our data. The top two plots are before removal of error and show the loci with their error rates in the DCH plot. The bottom plots are after removal of the error as evident from the DCH plot that shows no new individual added at any loci after the error removal.
