# Supplementary material for: Spatial genetic analysis reveals high connectivity of tiger (Panthera tigris) populations in the Satpura–Maikal landscape of Central India
Source: Ecol Evol. 2013 Jan 10;3(1):48–60. doi: 10.1002/ece3.432 (PMC3568842; doi:10.1002/ece3.432)
Supplement: Supplementary file 7 [file ece30003-0048-SD3.pdf]

Table S1. Information about climatic and vegetation attributes of tiger reserves in the Satpura-Maikal landscape.

**Kanha TR**	**Geocoordinates**	lat 22º 01 to 22º 28 N and long 80º 26 to 81º 04 E.
	**State**	Madhya Pradesh
	**Area (sq. kms)**	2059
	**Altitude (m)**	450-950
	**Rainfall (mm)**	1224
	**Temp (º C)**	-2 to 43
	**Vegetation**	Moist peninsular sal forest, southern tropical moist mixed deciduous forest, southern tropical dry deciduous mixed forest
**Pench TR (MP)**	**Geocoordinates**	lat 21º 37 to 21º 57 N and long 78º 56 to 79º 32 E
	**State**	Madhya Pradesh
	**Area (sq. kms)**	758
	**Altitude (m)**	400-650
	**Rainfall (mm)**	1400
	**Temp (º C)**	3-47
	**Vegetation**	Southern Indian tropical moist deciduous forest, southern tropical dry deciduous teak-bearing forest, southern dry mixed deciduous forest
**Pench TR (Mah.)**	**Geocoordinates**	lat 21º 32 to 21º 43 N to long 79º 04 to 79º 20 E
	**State**	Maharashtra
	**Area (sq. kms)**	258
	**Altitude (m)**	320-574
	**Rainfall (mm)**	1400
	**Temp (º C)**	14-43
	**Vegetation**	Southern tropical dry deciduous teak-bearing forest, southern dry mixed deciduous forest
**Bori-Satpura TR**	**Geocoordinates**	lat 22º 10 to 22º 43 N and long 77º 54 to 78º 34 E
	**State**	Madhya Pradesh
	**Area (sq. kms)**	1428
	**Altitude (m)**	320-1352
	**Rainfall (mm)**	1000-2200
	**Temp (º C)**	11-42
	**Vegetation**	South Indian moist teak forest, south Indian slightly moist teak forest, south Indian moist mixed forest, southern tropical dry teak forest, southern tropical dry mixed deciduous forest, dry penisular sal forest, central Indian subtropical hill forest
**Melghat TR**	**Geocoordinates**	21º 14 to 21º 45 N and 76º 53 to 77º 32 E
	**State**	Maharashtra
	**Area (sq. kms)**	1677
	**Altitude (m)**	350-1178
	**Rainfall (mm)**	1500-2200
	**Temp (º C)**	6-43
	**Vegetation**	Southern tropical dry deciduous forest
